# Voltage-dependent K^+^ channels as oncotargets in malignant gliomas

**DOI:** 10.18632/oncotarget.514

**Published:** 2012-06-01

**Authors:** Florence Lefranc, Henri-Benjamin Pouleau, Michal Rynkowski, Olivier De Witte

**Affiliations:** Department of Neurosurgery, Hôpital Erasme, Université Libre de Bruxelles (ULB), Brussels, Belgium; Department of Neurosurgery, Hôpital Erasme, Université Libre de Bruxelles (ULB), Brussels, Belgium; Department of Neurosurgery, Hôpital Erasme, Université Libre de Bruxelles (ULB), Brussels, Belgium; Department of Neurosurgery, Hôpital Erasme, Université Libre de Bruxelles (ULB), Brussels, Belgium

Gliomas account for more than 50% of all brain tumors and are by far the most common primary brain tumor in adults, and they are characterized by dismal prognoses, with glioblastoma representing the ultimate grade of malignancy [1]. The current standardized protocol to combat malignant gliomas includes maximal surgical resection followed by radiotherapy and temozolomide-based chemotherapy [2]. This standardized protocol had enabled the 5-year survival of glioblastoma patients to be increased from 2 to 10% [2]. However, malignant gliomas continue to have dismal prognoses, and this result is mainly due to two biological characteristics: i) malignant gliomas diffusely invade distant brain tissue through multiple single migrating cells, which limits the benefit of surgical resection; and ii) these migrating glioma cells resist pro-apoptotic stimuli, including conventional radio- and chemotherapy [1].

Reducing the levels of malignant glioma cell motility can restore glioma cell sensitivity to pro-apoptotic stimuli, and antimigratory compounds should therefore be added to conventional radio- and/or chemotherapy to restore certain levels of apoptotic sensitivity in glioma cells [1].

A growing body of work suggests that cellular migration and invasion in cancer cells in general and glioma cells in particular are facilitated by ion channels and transporters [3]. Glioma cells appear to shrink their cell volume to fit into the narrow extracellular spaces available and invade the brain parenchyma [3]. These features include the secretion of Cl^−^ and K^+^ through ion channels localized to lipid raft domains on invadipodia and water passively flowing through water channels or aquaporins [3]. In addition, the interplay between Na^+^-K^+^-Cl^−^ cotransporters and Na^+^/K^+^ ATPase lead to the active accumulation of K^+^ and Cl^−^, establishing a gradient for KCl efflux [3]. Pharmacological inhibition of large-conductance calcium-activated potassium channels or chloride channels impairs glioma cell migration and limits tumor progression in experimental models [3]. One Cl^−^ channel inhibitor, TM-601, a synthetic version of the peptide chlorotoxin, a small 36 amino acid neurotoxin isolated from the venom of the giant yellow Israeli scorpion *Leiurus quinquestriatus*, covalently linked to iodine 131 has completed Phase I and II clinical trials for the treatment of high-grade gliomas through repeated local intracavitary administrations [3]. An important number of studies have also reported the involvement of potassium channels in cancer progression. The largest family of potassium channels is the voltage-dependent potassium (Kv) channels. In this group, Kv1.3 and Kv1.5 channels modulate the proliferation of different mammalian cells, are involved in Ca^2+^ signaling and cell volume and have been analyzed in a number of cancer cell types [4]. The levels of expression of Kv1.5 and Kv1.3 channel subtypes discriminate between various glioma groups, and a clear differential expression of Kv1.5 is observed according to malignancy grade [5]. In addition, it has been shown that compared with normal cells, several human cancers have high mitochondrial membrane potential and low expression of the Kv1.5 channel, which both contribute to the apoptotic resistance of cancer cells, including gliomas [6]. Closing K^+^ channels or decreasing their expression in cancer cells results in an increase in intracellular K^+^ concentration, which in turn increases the tonic inhibition that cytosolic K^+^ exerts on caspases. As a result, Kv1.5 gene transfer directly activates apoptosis in apoptotic-resistant non-small lung cancer cells [6]. In addition, the functional inhibition of all Kv channels with 4-amino-pyridine limits dichloroacetate-induced apoptosis by 38% in glioblastoma cells, suggesting that although the majority of apoptosis in dichloroacetate-treated cells is a direct result of efflux of proapoptotic mediators from cancer cells, the secondary effects on Kv channels also play important roles [6].

Kv10.1 is virtually absent from normal cells outside the central nervous system and is frequently expressed in tumors, in which ectopic expression occurs in 70% of human cancers [7]. The targeted inhibition of Kv10.1 expression in cancer cells decreases their proliferation rate [7].

Several compounds already used in clinics could therefore be added to the conventional treatments used to combat malignant gliomas. Imipramine (Tofranil®), for example, is a well-known tricyclic antidepressant that binds to the intracellular Kv10.1 pocket and consequently inhibits its current [7]. Imipramine has already been demonstrated to reduce the proliferation, inhibit the PI3K/Akt/mTOR signaling and induce autophagic cell death in human glioma cells [8]. Citalopram (Cipramil®), which is a selective serotonin reuptake inhibitor used for its antidepressive activity, acts on Kv1.5 currents as an open-channel blocker.

Because both imipramine and citalopram have been commonly used to treat depression, which commonly occurs in glioma patients, it would be interesting to conduct large epidemiological studies to investigate the actual benefit that these two drugs would provide for malignant glioma patients when delivered during their glioma treatment. Such large-scale epidemiological studies have recently revealed the actual benefit provided by digoxin, a Na^+^/K^+^ ATPase inhibitor used to treat heart failure, in prostate cancer patients treated for both prostate cancer and heart failure [9].
